# How the Presence of a Doctor Known to Patients Impacts a Web-Based Intervention to Promote Physical Activity and Healthy Eating Behaviour in Individuals with an Overweight/Obesity–Hypertension Phenotype: A Randomised Clinical Trial

**DOI:** 10.3390/nu15071624

**Published:** 2023-03-27

**Authors:** Marta Ruiz-Cortés, Pedro Múzquiz-Barberá, Rocío Herrero, María Dolores Vara, Tamara Escrivá-Martínez, Raquel Carcelén, Enrique Rodilla, Rosa María Baños, Juan Francisco Lisón

**Affiliations:** 1 Department of Biomedical Sciences, Faculty of Health Sciences, University CEU-Cardenal Herrera, CEU Universities, 46115 Valencia, Spain; 2 Department of Nursing and Physiotherapy, Faculty of Health Sciences, University CEU-Cardenal Herrera, CEU Universities, 46115 Valencia, Spain; 3 Department of Psychology and Sociology, Universidad de Zaragoza, 50009 Teruel, Spain; 4 Centre of Physiopathology of Obesity and Nutrition (CIBERobn), CB06/03/0052, Instituto de Salud Carlos III, 28029 Madrid, Spain; 5 Polibienestar Research Institute, Universitat de València, 46022 Valencia, Spain; 6 Department of Medicine and Surgery, Faculty of Health Sciences, University CEU-Cardenal Herrera, CEU Universities, 46115 Valencia, Spain; 7 Hypertension and Vascular Risk Unit, Hospital Universitario de Sagunto, 46115 Valencia, Spain

**Keywords:** e-health, physical activity, Mediterranean diet, eating behaviour, motivation, obesity, overweight, hypertension

## Abstract

(1) Background: The ‘Living Better’ web-based programme has shown short- and long-term benefits for body composition and psychological variables in obese patients with hypertension by promoting a healthier lifestyle. To further explore the potential of this programme, in this work we aimed to explore the possible effect of the patient’s ‘own doctor’ appearing in the video content of the Living Better intervention. (2) Methods: A total of 132 patients were randomly assigned either to the experimental (EG, *n* = 70) or control (CG, *n* = 62) group (with a doctor the patient knew as ‘their own’ or an ‘unknown doctor’, respectively). The body mass index (BMI), motivation towards physical activity (PA), PA levels, motivation to change one’s eating habits, adherence to the Mediterranean diet, and eating behaviour were all assessed and compared at baseline and post-intervention (12 weeks). (3) Results: The results of this study confirmed the positive effects of the Living Better programme on BMI and external eating style, with significant improvements in these variables in both groups. In addition, in the EG there was higher intrinsic motivation to change eating behaviour (mean difference of 0.9, 95% CI [0.1, 1.6], *p* = 0.032) and lower amotivation (mean difference of −0.6, 95% CI [−1.2, −0.1], *p* = 0.027) compared to the CG. (4) Conclusions: This study suggests that the presence of the patients’ own doctor in the audiovisual content of the Living Better intervention did not have significant additional benefits in terms of BMI or external eating style. However, their presence did improve intrinsic motivation and amotivation related to eating habits.

## 1. Introduction

The prevalence of obesity has increased nearly threefold over the past few decades, creating a significant burden not only on individuals’ health but also on society as a whole. Obesity has become a global pandemic [1,2], leading to a rise in related conditions such as cardiovascular disease (CVD), type 2 diabetes, fatty liver disease, dementia, osteoarthritis, obstructive sleep apnea, and various forms of cancer [1,3]. In fact, CVD is the leading cause of mortality in people with obesity, accounting for approximately 70% of deaths in this population [1]. Hypertension is one of the established risk factors contributing to the increased CVD risk among obese individuals [4]. Therefore, the continuous study of new therapeutic approaches for the treatment of obesity and/or hypertension is warranted.

The international guidelines specialising in obesity [5] and hypertension [6,7] agree that the first step to consider in clinical approaches to patients with obesity and/or hypertension should be the promotion and acquisition of a healthier lifestyle, based on two key pillars: the establishment of healthy eating behaviours and regular engagement in physical activity (PA). Likewise, with the aim of promoting proactive disease control by patients and reducing the burden of care, for many years now the World Health Organization [8] has been trying to encourage health interventions administered through the internet and technologies. Some of the strengths of this type of intervention are their cost-effectiveness, accessibility (overcoming barriers and limitations, for example, during confinement), flexibility (in terms of when and where they can be completed, etc.), and their potential for personalisation [9,10,11].

Several systematic reviews and meta-analyses have examined the use of technology and the internet in treating obesity and hypertension and have found that online interventions promoting healthy lifestyles can effectively improve body weight and/or blood pressure levels [12,13,14,15]. However, there has been limited research on the effectiveness of these treatments for patients with both conditions, specifically those with an obesity–hypertension phenotype [16,17,18,19,20]. In the most recent research in this area, our group specifically designed and applied an online intervention programme called ‘Living Better’, which aimed to improve the body composition of obese patients with hypertension by promoting a healthier lifestyle [18,19,20]. This 3-month programme, which includes extensive multimedia content, is based on psychoeducation and promotes regular engagement in PA as well as the establishment of healthy eating habits. We applied this multidisciplinary, multimedia, interactive, and self-administered intervention in an experimental context and reported significant improvements in body composition and psychological variables such as anxiety, stress, and eating behaviours, and most importantly, the participants maintained positive long-term (3-year) health benefits [20].

To take this research further, in this current study we aimed to explore the possible effect of the patients’ ‘own doctor’ appearing in all the videos of the Living Better programme. Using video modelling, which involves the demonstration of desired behaviours, outcomes, and attitudes through active visual representations by an actor, is considered an effective way to educate and guide patients through behavioural interventions, even among people with low levels of literacy [21]. Furthermore, it has been shown that the simple gesture of doctors talking to patients about their own personal practices in terms of PA and nutrition helps to promote general patient health. This is because patients are more likely to adopt healthy behaviours when their doctor also practices them (referred to as the ‘lead by example’ practice [22]. Indeed, the therapeutic alliance, understood as the quality of the relationship between the patient and the therapist, seems to be a decisive factor in patients assuming more proactive roles in their own health care [23,24].

Therefore, although to date we are not aware of previous studies that have analysed the possible effect of the audiovisual presence of a therapist (psychologist, doctor, nurse, physiotherapist, or nutritionist, etc.) in online interventions designed to prevent or treat diseases, it is plausible that their presence may improve patients’ motivation to change their eating habits and attitudes towards PA. This change would also bring about a change in lifestyle itself (eating habits and PA levels) and by extension, body composition. Considering all the above, the aim of this present research was to analyse the influence exerted by the identity of the main doctor appearing in the audiovisual content of the Living Better web-based intervention on patients with the obesity–hypertension phenotype in terms of the following variables: body mass index (BMI), PA levels, adherence to the Mediterranean diet, motivation towards PA, motivation to change eating habits, and eating style. We hypothesised that patients who saw their own doctor giving them the indications would attain greater benefits in the different variables analysed than those in the ‘unknown doctor’ control group.

## 2. Materials and Methods

### 2.1. Study Design

The present study was a prospective, single-centre, randomised clinical trial that followed the ethical guidelines established in the Declaration of Helsinki and was approved by the Hospital of Sagunto Human Ethics Committee. The trial was registered at ClinicalTrials.gov (NCT04739033) and conducted according to the details outlined in the CONSORT statement [25]. Balanced randomisation (1:1) was used to assign participants to the control and experimental groups.

#### Eligibility Criteria

Eligible participants were all adults/older adults aged between 18 and 75 years with hypertension and who were overweight (BMI > 24.9 kg/m^2^ and < 30 kg/m^2^) or had type 1 obesity (BMI > 29.9 kg/m^2^ and < 35 kg/m^2^), and who were patients managed by the same physician specialised in the obesity–hypertension phenotype. Hypertension was defined as a systolic blood pressure ≥ 140 mmHg and/or a diastolic blood pressure ≥ 90 mmHg, or the current use of antihypertensive medication. Regarding the exclusion criteria, patients who had not come for at least 1 visit with their specialist in the 5 years prior were excluded from the trial. In addition, profiles with previous ischemic heart disease, cerebrovascular disease, severe psychiatric disorders, taking more than 3 antihypertensive drugs, with physical impairments precluding participation in PA, receiving any treatment for weight loss elsewhere, who had previously participated in the Living Better intervention [18,19,20], and/or without internet access were also excluded from this current study.

### 2.2. Procedure

**Figure 1:**
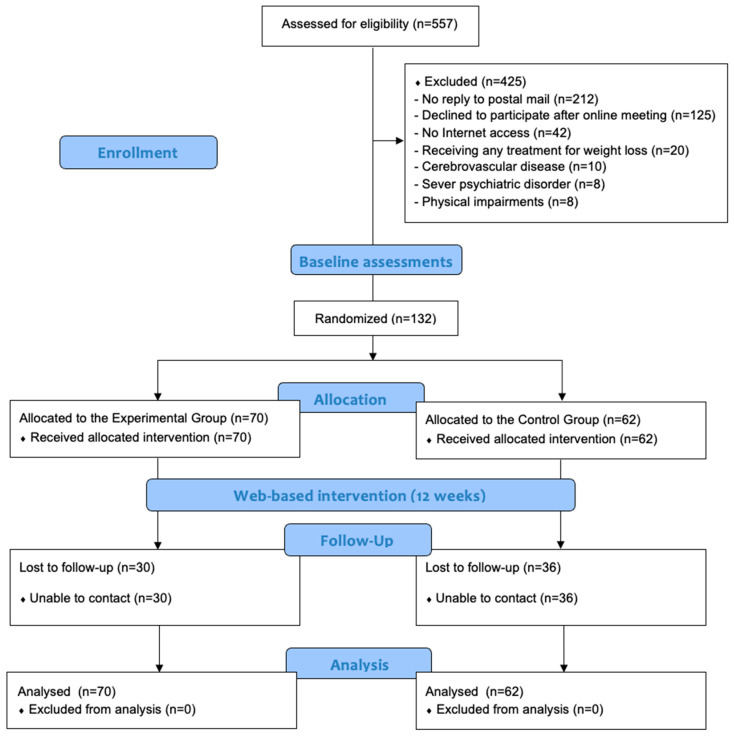
Progression of the participants through the trial.

This work took place at the Hospital Universitario de Sagunto (Valencia, Spain) between February and June 2021. All participants from the hospital’s Hypertension and Vascular Risk Unit with the obesity–hypertension phenotype who had not previously participated in the Living Better studies [18,19,20] were invited to participate by postal mail (*n* = 557). All those who met the inclusion/exclusion criteria and agreed to participate after an online meeting had to read and sign an informed consent to participation before being enrolled in the study (*n* = 132). At the beginning of the trial, a researcher (researcher 1) who was unaware of the study characteristics created a random sequence using a computerized random number generator. This sequence was concealed from all other study investigators throughout the entire study period. Randomization was stratified for the number of specialist visits, age, and sex. Participants were enrolled in the study and completed baseline outcome measures before being randomly assigned to either the control group (CG; *n* = 62) or the experimental group (EG; *n* = 70). Although it was not possible to mask the group allocation from the participants, the outcome assessors and data analysts were blinded to the treatment allocations. As shown in the participant flowchart (Figure 1), the different study variables were recorded at baseline (just before the start of the intervention) and immediately after having completed the 12-week Living Better web-based programme.

### 2.3. Intervention

Living Better is a computerised intervention that is self-administered through the internet. The treatment protocol consists of 9 modules and incorporates psychological strategies that encourage a healthy lifestyle by progressively establishing healthy eating habits and increasing the level of PA, as recommended by international guidelines. A period of 12 weeks was allowed for the completion of the entire programme, during which time the modules were activated weekly or fortnightly. Some of the techniques used were self-monitoring, self-instruction, behavioural recording, stimulus control, self-reinforcement, problem-solving techniques, and homework. In addition, the accompanying web page (designed and hosted by a web-based platform called Wix) offered useful tools such as the ability to download documents online and view multiple videos and had a responsive design that adjusted the site for viewing with mobile devices. More details about the intervention can be found in Baños et al. [26], Mensorio et al. [18], Lison et al. [19], and Múzquiz-Barberá et al. [20].

The programme content followed by both groups was identical, with the exception that the physician who appeared in the audiovisual material differed between them; the CG patients saw a doctor that they did not know while those assigned to the EG saw their own obesity–hypertension phenotype specialist. Specifically, Living Better contains 32 videos (reaching a total of 52 min) of the presenting doctors’ audiovisual presence. Both the doctors involved in delivering the audiovisual content in this study were specialists in the obesity–hypertension phenotype and vascular risk, regularly engaged in PA, and had a healthy appearance.

### 2.4. Outcome Measures

The patient’s age, sex, and the number of visits to the specialist were all registered before the randomisation process was implemented. Furthermore, the variables listed below were recorded via the same platform as the intervention programme.

### 2.5. Primary Outcome

Because of the indications of the health authorities and hospital regulations related to the COVID-19 pandemic, the participants were instructed to register their BMI in a pharmacy near their homes. They were also instructed to go to the pharmacy while fasting to avoid the possibility that any food or drink ingested could have influenced their data. Thus, the same person (pharmacist or pharmacy assistant) used an approved device to assess their weight and height; BMI was calculated by dividing patient weight by their height squared (kg/m^2^).

### 2.6. Secondary Outcomes

Physical activity levels were evaluated using the abbreviated version of the International Physical Activity Questionnaire (IPAQ short form), which has been validated for use in the Spanish population [27,28]. This self-administered questionnaire includes 7 items that collect information on the number of days per week and minutes per day spent engaging in moderate or vigorous exercise, walking, or sedentary activities. Dietary habits were assessed using the Mediterranean Diet Adherence Screener (MEDAS) questionnaire from the PREDIMED study. This questionnaire, which has also been validated for the Spanish population, measures adherence to the Mediterranean diet, a dietary pattern that has been shown to be effective in preventing and reducing the incidence of cardiovascular diseases [29]. The MEDAS questionnaire includes 14 items, 12 of which assess the frequency of food consumption and the remaining 2 assess adherence to the characteristic dietary habits of the Spanish Mediterranean diet. Each item is scored with a 0 or 1, and based on the total score, participants are classified as having low adherence (score between 0–5), moderate adherence (score between 6–9), or high adherence (score ≥ 10) to the dietary pattern. The reproducibility of this questionnaire has been evaluated and found to be good [30].

Motivation towards physical activity (PA) was evaluated using the validated Spanish version of the Behavioural Regulation in Exercise Questionnaire-2 (BREQ-2; [31,32]), which consists of 19 items measuring stages on the self-determination continuum using a 5-point Likert scale. The BREQ-2 assesses five different subscales: intrinsic regulation (4 items), identified regulation (4 items), introjected regulation (3 items), external regulation (4 items), and amotivation (4 items), with a maximum score of 20 for intrinsic regulation, identified regulation, external regulation, and amotivation, and a maximum score of 15 for introjected regulation. Intrinsic regulation involves participating in an activity for inherent enjoyment and satisfaction. Identification involves consciously accepting the behaviour as important to achieve personally valued outcomes. Introjected regulation involves the internalisation of external controls, resulting in self-imposed pressures to avoid guilt or maintain self-esteem. External regulation involves engaging in behaviour solely to satisfy external pressures or achieve externally imposed rewards. Amotivation refers to a state of lacking the intention to engage in a behaviour and is a non-self-determined form of regulation [31]. Self-determined regulation towards PA, particularly intrinsic motivation, is related to higher adherence to PA [33,34,35,36]. This questionnaire was adapted to assess patient motivation to change eating habits [18].

Eating behaviour was assessed using the Dutch Eating Behaviours Questionnaire (DEBQ; [37], which has been validated for the Spanish population [38]. This comprises 33 items on a 5-point Likert scale that evaluate three styles of eating: emotional eating (13 items), restrictive/restrained eating (10 items), and external eating (10 items). Higher scores in each subscale reflect a higher level of emotional, restrictive/restrained, and external eating, respectively. Emotional eating consists of overeating in response to mainly negative emotional states for the purpose of regulating said state; external eating is conceptualised as a greater tendency to eat in response to external cues and a greater sensitivity to environmental cues than to internal cues, i.e., physiological hunger cues. In turn, restrictive eating refers to the tendency to restrict food intake or reduce the amount of food eaten to achieve weight loss or prevent weight gain. It is also known that these three intake styles may be associated with unhealthy eating and an increased BMI [39,40,41,42].

Finally, at the end of the intervention, the online platform automatically collected the degree of programme completion by each patient (number of modules they had reviewed out of a total of 9).

### 2.7. Statistical Analysis

A sample size of 50 patients per group was required to achieve a statistically significant 0.85-point BMI reduction between the estimated mean and the sampling mean, in line with previous study data [18], with a statistical power of 80% and an alpha risk of 0.05 [43]. The desired sample size was calculated by an external researcher not involved in the study procedures, who was blinded to the intervention. To compensate for potential participant dropouts and possible alterations in the statistical significance of the results, the sampling size was increased by 30%. The statistical analysis was performed according to the intention-to-treat principle. The Kolmogorov–Smirnov test was used to check compliance with the normality assumption for each dependent variable. The proper randomization was verified by Chi-squared and independent sample Student *t*-tests. An independent sample Student *t*-test was also performed to compare the number of modules reviewed between the two groups. Two-way (2 × 2) mixed analysis of variance (ANOVA) tests were used to compare the study effects on BMI, physical activity levels, adherence to the Mediterranean diet, motivation to change eating habits, motivation towards physical activity, and eating style, with time (baseline versus post-intervention) as the within-group factor and group (CG versus EG) as the between-group factor. Bonferroni post hoc tests were applied following the results of the ANOVA tests. The effect sizes (ηp2) were calculated, where 0.01 < 0.06, 0.06 < 0.14, and > 0.14 corresponded to a small, medium, and large effect size, respectively [44]. SPSS version 19.0 for Windows was used for statistical analyses and the statistical significance was set at *p* ≤ 0.05 for all analyses. The data were presented as means ± standard deviation (SD) in this study.

## 3. Results

We screened 557 participants in this randomised controlled trial. A total of 425 individuals were not allocated for randomisation because they did not respond to the invitation by postal mail (*n* = 212), declined to participate after the online meeting (*n* = 125), or did not meet the inclusion criteria (*n* = 88; Figure 1). Finally, 132 Caucasian adults participated in the study. The baseline characteristics of both groups were similar and no significant differences were observed (Table 1). Similarly, no significant differences were found between the number of modules reviewed by the participants of both groups (CG: 4.5 ± 2.7; EG: 4.5 ± 2.6, *p* = 0.991).

The results of the two-way ANOVA analyses showed significant time-by-group interaction effects for the intrinsic motivation to change eating habits and amotivation, with large effect sizes (ηp2 ≥ 0.032; Table 2).

**Figure 2:**
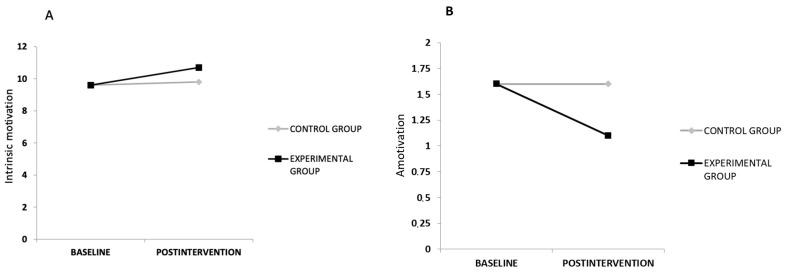
Pre- and post-differences between the experimental and the control groups in terms of intrinsic motivation (**A**) and amotivation (**B**) to change eating habits.

After adjusting the ANOVA results for baseline data, the post hoc tests showed higher intrinsic motivation to change eating habits (mean difference of 0.9, 95% CI [0.1, 1.6]; *p* = 0.032) and lower amotivation (mean difference of −0.6, 95% CI [−1.2, −0.1]; *p* = 0.027) in the EG compared to the CG (Figure 2). However, the two-way ANOVA results did not show significant time-by-group interaction effects in the rest of the study variables. In addition, the results of the ANOVA tests showed significant time effects for BMI and external eating behaviour, with significant within-group improvements after the interventions in these variables in both groups (BMI: EG, mean difference of −0.4, 95% CI [−0.6, −0.2], *p* < 0.001; CG, mean difference of −0.3 [−0.5, −0.1], *p* = 0.003; and external eating behaviour: EG, mean difference of −1.1 [−2.1, −0.1], *p* = 0.037; CG, mean difference of −1.4 [−2.4, −0.3], *p* = 0.014).

## 4. Discussion

This study analysed the influence of the audiovisual presence of patients’ own specialist doctor in an online intervention programme designed to promote a healthy lifestyle, adherence to the Mediterranean diet, motivation to change eating habits, eating behaviour, and motivation towards PA in patients with an obesity–hypertension phenotype. Our results confirmed the positive effects of the Living Better intervention on BMI [18,19,20] and external eating behaviour [18], with statistically significant improvements being observed in these variables in both study groups. Mensorio et al. [18] have previously reported the effectiveness of the Living Better program in modifying external eating behaviour. The program aimed to change eating behaviours by incorporating psychoeducation, eating tricks, and self-management strategies to generate a more conscious and less impulsive eating style. This finding is particularly relevant as eating styles are considered stable and dimensional factors that are closely linked to obesity [45]. However, contrary to our initial hypothesis, at the end of the intervention, the EG showed no significant differences in relation to the CG either in terms of BMI or external eating behaviour. However, the presence of the patient’s own doctor in the Living Better multimedia material did result in significant improvements in intrinsic motivation and amotivation related to eating habits compared to the CG. Moreover, motivation to change played a key role in the initiation and continued engagement in healthy eating behaviour. Nonetheless, these differences did not translate into differences between the two groups in terms of adherence to the Mediterranean diet.

Despite the above, it is worth noting that, regardless of which doctor appeared in the audio-visual material, Living Better still achieved Mediterranean dietary eating habits close to the upper limit of the average adherence range (above 8 points), even though the interventions took place during the COVID-19 confinement period. This is especially interesting given that some studies have demonstrated the negative effects the COVID-19 pandemic had on eating habits in general [46,47,48]. Thus, for example, Pietrobelli et al. [46] observed an increase in the consumption of processed foods such as crisps and sugary drinks in obese Italian children during this period. Similarly, Ghosh et al. [47] reported an increase in the frequency and consumption of snacks and carbohydrate-rich foods in diabetic Indian patients. Finally, a recent study revealed that vegetables, olive oil, fruits, nuts, legumes, and fish (foods that are characteristic of the Mediterranean diet) were notably under consumed among university students in Mediterranean and non-Mediterranean countries during the COVID-19 pandemic [48].

Regarding PA, as in previous studies [18,19,20], at the end of the intervention, there were no differences in PA levels between the groups, although both showed a slight trend towards improvement; this was in line with the improvement trends found in both groups in terms of motivation towards PA (external motivation and amotivation). It is possible that the high levels of PA at the start of the study (above 3000 metabolic equivalents [METs] in the EG) contributed to the lack of significant results. In general, most studies evaluating online interventions for the obese and hypertensive population have not considered baseline levels of PA and, when considered, the results were inconclusive. In this sense, it is generally thought that this variable is not easy to change [49]. It is also important to note that the maintenance or slight increase in PA levels seen during the intervention should be interpreted as a positive result. In this regard, it is also worth bearing in mind that just before the start of this work (28 October 2020) a second state of national alarm with movement restrictions was declared in Spain [50] and a third alarm state coincided with the elaboration of this present study [51]. Interestingly, a recent meta-analysis with a total sample size of 119,094 participants from 14 countries worldwide and with participants aged between 4 and 93 years also revealed a significant decline in PA in all age groups during this time, independently of gender [52]. Another meta-analysis concluded that, during the COVID-19 pandemic, PA levels were also reduced in patients with chronic diseases with respect to their previous levels [53].

In this current study, the number of losses in the post-intervention assessments was manifestly higher compared to those from the previous studies [18,19,20]. This may be the result of participant difficulties in the context of the ongoing COVID-19 pandemic (for example, the post-intervention measurements had to be conducted outside of the hospital context because of the health restrictions resulting from COVID-19). Remarkably, despite the increased dropout rate, the results of the intention-to-treat statistical analysis showed that the participants of both groups experienced significant health benefits. It is worth noting that the internet has been proven to be an effective means of preventing and treating chronic diseases by promoting healthy lifestyles. One of the main advantages of internet-based interventions is that they can reach a larger population, including those with limited access to health services or social support, and can provide more intensive contact at potentially lower costs than conventional face-to-face programs [54,55,56,57]. Additionally, internet-based interventions can offer immediate, easily accessible, one-to-one, and ongoing support, as well as individually tailored care, to encourage behavioural change. Patients can receive care in the comfort of their own homes with self-paced delivery [56,58,59]. Interestingly, all these advantages are of particular interest in times of a pandemic because they provide health workers with effective therapeutic tools that can address the barriers imposed by the restrictions associated with confinement.

The present study had some limitations worth mentioning. Firstly, participants who enrolled in the study had already demonstrated an initial level of motivation towards engaging in an e-health programme, which may have introduced selection bias. Therefore, our findings may only be applicable to individuals with internet access who are similarly interested in e-health interventions [60]. Additionally, our participants were recruited from a public hospital instead of a private one, which may have influenced our results because sociodemographic status has been linked to treatment adherence for chronic conditions [61,62]. Secondly, recall bias may have affected the study’s results, as all participants’ responses to the questionnaires depended on their ability to remember their habits accurately. Similarly, desirability bias may have occurred, where participants tended to downplay unhealthy habits and exaggerate healthy behaviours [63]. Thirdly, this single-centre clinical trial only involved one doctor per arm and, therefore, was potentially confounded by the personal characteristics of these doctors that could have influenced the outcomes. Finally, the dropout rate was high which might have limited the within- and between-group differences. Although this finding is frequently observed in online interventions, it is still worth discussing as it presents a significant obstacle to their broader adoption [64,65].

## 5. Conclusions

Our findings suggest that the presence of patients’ own doctor in the audiovisual content of an online intervention programme aimed at promoting a healthy lifestyle did not show significant additional benefits in terms of BMI, levels of physical activity, adherence to the Mediterranean diet, eating behaviour, or motivation towards physical activity among patients with an obesity-hypertension phenotype. However, their presence did improve intrinsic motivation and amotivation related to eating habits. Future studies with multiple doctors per arm and with a larger and more representative sample size should be performed to investigate the short- and long-term impact of the presence of patients’ own doctor in such audiovisual web-based interventions for adults with this phenotype.

## Figures and Tables

**Table 1:** Baseline characteristics of the study participants.

	Group		
	Control (*n* = 62)	Experimental (*n* = 70)	*p*
Age (years)	57.7 (10.7)	56.2 (9.5)	0.397
Men/women (*n*)	34/28	38/32	0.949
Specialist visits (*n*)	9.8 (8.3)	9.4 (7.9)	0.819
BMI	29.7 (3.2)	29.5 (3.6)	0.787
Physical activity (IPAQ) METs	2692 (3299)	3046 (3220)	0.542
Adherence to the Mediterranean diet (MEDAS)	8.1 (2.0)	8.4 (2.0)	0.388
Motivation to change eating habits			
Intrinsic	9.5 (3.9)	9.7 (4.4)	0.857
Identified	9.8 (2.4)	10.1 (2.1)	0.463
Introjected	5.6 (3.0)	6.5 (3.4)	0.145
External	3.5 (3.8)	4.0 (3.9)	0.476
Amotivation	1.6 (2.9)	1.8 (3.1)	0.706
Motivation towards PA			
Intrinsic	10.3 (4.2)	10.8 (4.6)	0.477
Identified	10.5 (2.7)	11.2 (3.2)	0.245
Introjected	5.6 (3.6)	5.4 (3.6)	0.662
External	4.7 (4.1)	3.8 (3.9)	0.194
Amotivation	2.5 (3.5)	2.0 (2.9)	0.461
Eating style (DEBQ)			
Emotional	29.5 (12.4)	30.3 (13.9)	0.745
Restrictive	26.0 (8.1)	27.4 (8.3)	0.332
External	28.6 (7.6)	27.7 (8.8)	0.551

Data presented as mean (SD).

**Table 2:** Results of the 2 × 2 ANOVA tests.

	Group				ANOVA Effects						
	Control (*n* = 62)		Experimental (*n* = 70)		Time		Group		Time × Group		
	Pre	Post	Pre	Post	*F*	*p*	*F*	*p*	*F*	*p*	ηp2
BMI	29.7 (3.2)	29.4 (3.5)	29.5 (3.6)	29.1 (3.6)	26.3	0.000	0.118	0.732	0.481	0.489	0.004
Physical activity (IPAQ) METS	2692 (3299)	2931 (3049)	3046 (3220)	3429 (3179)	3.40	0.068	0.620	0.433	0.182	0.670	0.001
Adherence to the Mediterranean diet (MEDAS)	8.1 (2.0)	8.5 (2.1)	8.4 (2.0)	8.5 (2.1)	7.44	0.007	0.236	0.628	2.04	0.156	0.017
Motivation to change eating habits											
Intrinsic	9.5 (3.9)	9.8 (4.1)	9.7 (4.4)	10.8 (3.9)	11.1	0.001	0.596	0.442	3.92	0.050	0.032
Identified	9.8 (2.4)	9.9 (2.5)	10.1 (2.1)	10.6 (1.9)	2.99	0.086	1.74	0.189	2.54	0.114	0.021
Introjected	5.6 (3.0)	5.7 (3.2)	6.5 (3.4)	6.7 (3.5)	0.741	0.391	2.81	0.096	0.134	0.715	0.001
External	3.5 (3.8)	3.0 (3.6)	4.0 (3.9)	3.7 (3.9)	1.57	0.212	0.894	0.346	0.152	0.697	0.001
Amotivation	1.6 (2.9)	1.6 (2.9)	1.8 (3.1)	1.1 (2.3)	4.43	0.037	0.082	0.775	4.43	0.037	0.036
Motivation towards PA											
Intrinsic	10.3 (4.2)	10.4 (4.4)	10.8 (4.6)	10.6 (4.5)	0.016	0.899	0.228	0.634	0.981	0.324	0.008
Identified	10.5 (2.7)	10.8 (2.8)	11.2 (3.2)	11.4 (2.8)	2.83	0.095	1.51	0.220	0.006	0.940	0.000
Introjected	5.6 (3.6)	5.9 (3.5)	5.4 (3.6)	5.3 (3.6)	0.141	0.708	0.546	0.462	0.506	0.478	0.004
External	4.7 (4.1)	4.1 (4.0)	3.8 (3.9)	3.3 (3.9)	4.86	0.029	1.83	0.178	0.017	0.898	0.000
Amotivation	2.5 (3.5)	2.0 (3.2)	2.0 (2.9)	1.7 (2.7)	4.19	0.043	0.424	0.516	0.203	0.653	0.002
Eating style (DEBQ)											
Emotional	29.5 (12.4)	28.7 (12.0)	30.3 (13.9)	29.9 (14.3)	1.37	0.243	0.180	0.672	0.150	0.699	0.001
Restrictive	26.0 (8.1)	26.0 (8.4)	27.4 (8.3)	27.4 (6.9)	0.000	0.985	1.11	0.293	0.000	0.985	0.000
External	28.6 (7.6)	27.2 (8.2)	27.7 (8.8)	26.6 (8.5)	10.7	0.001	0.265	0.608	0.155	0.695	0.001

Data presented as mean (SD).

## Data Availability

The data that support the findings of this study are available from the corresponding author upon reasonable request.
